# Pentraxin 3 deficiency enhances features of chronic rejection in a mouse orthotopic lung transplantation model

**DOI:** 10.18632/oncotarget.23902

**Published:** 2018-01-03

**Authors:** Mitsuteru Yoshida, Hisashi Oishi, Tereza Martinu, David M. Hwang, Hiromitsu Takizawa, Junichi Sugihara, Trevor D. McKee, Xiaohui Bai, Zehong Guana, Christina Lua, Hae-Ra Cho, Stephen Juvet, Marcelo Cypel, Shaf Keshavjee, Mingyao Liu

**Affiliations:** ^1^ Latner Thoracic Surgery Research Laboratories, Toronto General Hospital Research Institute, University Health Network, Toronto, Ontario, Canada; ^2^ Institute of Medical Science, University Health Network, Toronto, Ontario, Canada; ^3^ Department of Medicine, University Health Network, Toronto, Ontario, Canada; ^4^ Laboratory Medicine and Pathobiology, University Health Network, Toronto, Ontario, Canada; ^5^ Surgery, Faculty of Medicine, University of Toronto, Toronto, Ontario, Canada

**Keywords:** innate immunity, Micro-CT, bronchiolitis obliterans, lymphatic aggregates, Th1/Th2 response

## Abstract

Chronic lung allograft dysfunction (CLAD) is a serious complication after lung transplantation and thought to represent chronic rejection. Increased expression of Pentraxin 3 (PTX3), an acute phase protein, was associated with worse outcome in lung transplant patients. To determine the role of recipient PTX3 in development of chronic rejection, we used a minor alloantigen-mismatched murine orthotopic single lung transplant model. Male C57BL/10 mice were used as donors. Male PTX3 knockout (KO) mice and their wild type (WT) littermates on 129/SvEv/C57BL6/J background were used as recipients. In KO recipients, 7/13 grafted lungs were consolidated without volume recovery on CT scan, while only 2/9 WT mice showed similar graft consolidation. For grafts where lung volume could be reliably analyzed by CT scan, the lung volume recovery was significantly reduced in KO mice compared to WT. Interstitial inflammation, parenchymal fibrosis and bronchiolitis obliterans scores were significantly higher in KO mice. Presence of myofibroblasts and lymphoid aggregation was significantly enhanced in the grafts of PTX3 KO recipients. Recipient PTX3 deficiency enhanced chronic rejection-like lesions by promoting a fibrotic process in the airways and lung parenchyma. The underlying mechanisms and potential protective role of exogenous PTX3 as a therapy should be further explored.

## INTRODUCTION

Chronic lung allograft dysfunction (CLAD), with an incidence of approximately 50% at 5 years after lung transplantation, is the biggest impediment to long-term survival [1]. Damage to the airway epithelium and remodeling of the small airways with fibrotic obliteration and fibrosis of the lung parenchyma are major pathological features of CLAD [2]. Chronic rejection, through allogeneic immune response-induced lymphatic infiltration and lymphoid tissue formation in the lung grafts, is considered the most important contributing factor [3–6]. Increasing evidence suggests that inflammation mediated by innate immune responses during ischemia-reperfusion in the lung allograft [7, 8] and activation of autoimmunity [9] are also involved in the pathogenesis of CLAD. For example, primary graft dysfunction (PGD), acute lung injury during the first 72 h after lung transplant, is associated with the development of CLAD [10–12]. Identification of biomarkers of PGD may help to predict, and possibly prevent, PGD as well as CLAD.

Diamond et al. reported that plasma levels of PTX3, an acute phase protein, were significantly increased after lung transplantation, and were associated with the development of PGD in transplant recipients with idiopathic pulmonary fibrosis (IPF), but not in those with COPD [13]. The same group also reported that two genetic variants of recipient PTX3 are associated with the development of PGD, and one of the variants is associated with increased PTX3 plasma levels [14]. Changes in PTX3 expression have also been reported in kidney transplantation whereby PTX3 expression positively correlated with the degree of allograft dysfunction and acute renal allograft rejection [15].

PTX3 is an important inflammatory mediator and a critical component of the humoral arm of innate immunity [16, 17]. PTX3 is a soluble pattern recognition receptor with both immunostimulatory and immunoregulatory functions. It orchestrates clearance of pathogens through opsonization of damage- and pathogen-associated molecular patterns, regulates clearance of apoptotic and necrotic cells, and prevents autoimmune pathology [18, 19]. Elevation of PTX3 levels in the circulation following infection or tissue damage appears to predict poor patient outcome [19], as seen in patients with sepsis and acute lung injury [20–22].

On the other hand, it has been shown that PTX3 provides protection against specific fungal, bacterial or viral pathogens [18, 19]. Following allogeneic transplantation, reactivation of latent human cytomegalovirus is a major cause of morbidity and mortality and predisposes to severe complications, including superinfection by *Aspergillus* species [23]. In a murine model of allogeneic bone marrow transplantation, PTX3 treatment induced complete resistance to infection and reinfection with invasive aspergillosis [24]. PTX3 was shown to bind cytomegalovirus, and protected mice from primary viral infection and reactivation as well as *Aspergillus* superinfection [23]. Genetic deficiency of PTX3 in humans impairs the antifungal capacity of neutrophils and may contribute to the risk of invasive aspergillosis in patients treated with hematopoietic stem-cell transplantation [25].

The function of PTX3 in solid organ transplantation, however, has not been studied. In the present study, we hypothesized that the endogenous recipient-derived PTX3 plays an important role in chronic rejection pathogenesis and dampens inflammation and fibrosis after lung transplantation. We tested our hypothesis using a minor-mismatched orthotopic lung transplantation model [26] with PTX3 knockout (KO) mice and their wild type (WT) littermates as recipients.

## RESULTS

### PTX3 KO recipients show increased lung graft consolidation and reduced lung volume

We set off to perform left lung transplantation on WT and KO mice. Three KO recipients died early after lung transplantations on days 3 (*n* = 2) and 10. These mice were found up to 16 hours after death and the autopsy did not identify a cause of death. Due to this early mortality and more lung consolidation (see below), more KO mice were transplanted, for a total of 9 WT and 13 KO recipients that survived until day 28.

**Figure 1:**
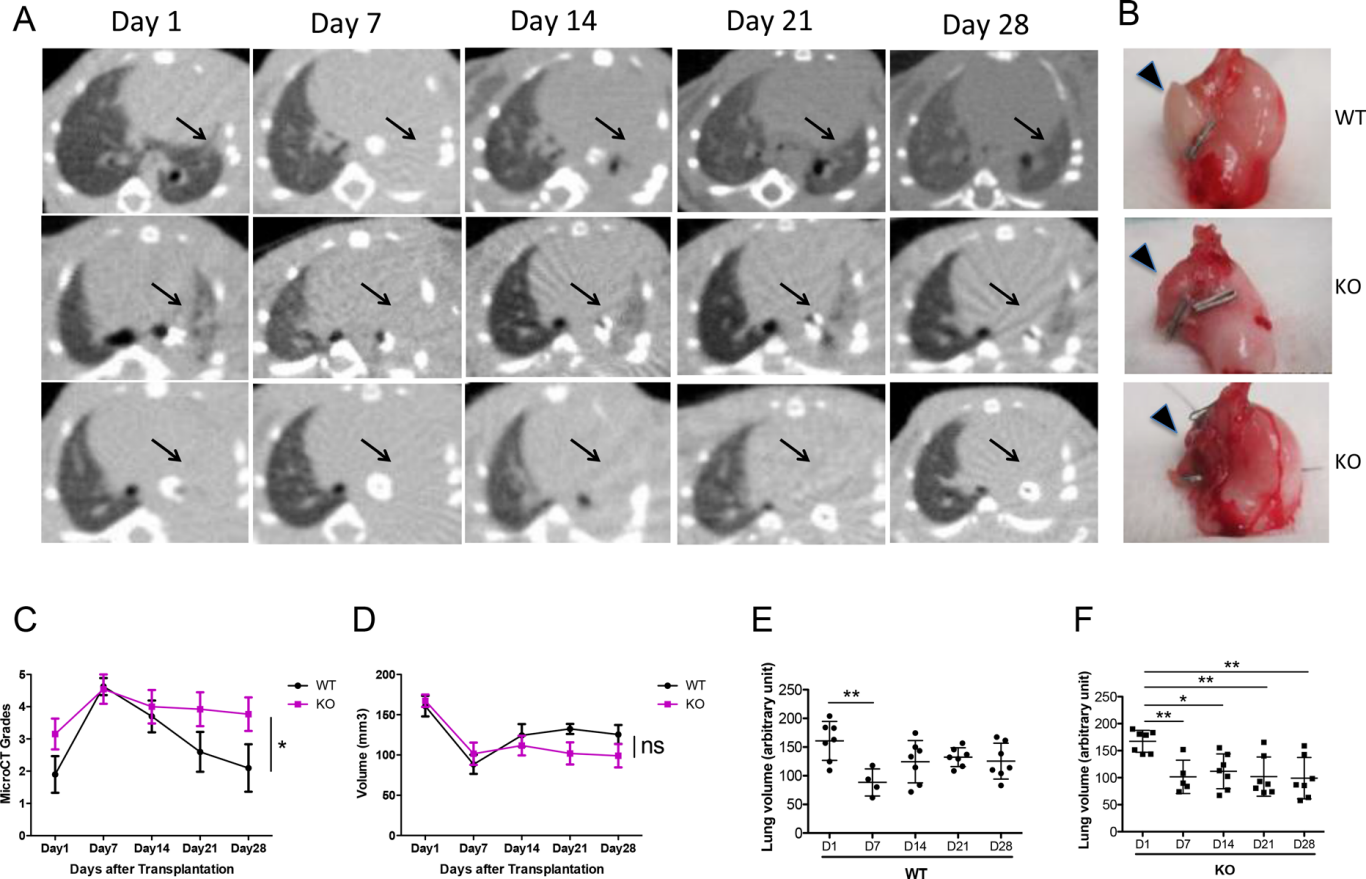
Increased consolidation of lung grafts in PTX3 KO mice (**A**) Sequential micro-CT scans were performed after lung transplantation. Transplanted left lung grafts are indicated with arrows. Almost all recipients show left lung consolidation on Day 7 after lung transplantation. Aeration of the graft lung recovered in 7 out of 9 WT recipients (top panel) and in 6 out of 13 KO recipients (middle panel), and did not recover in other KO recipients (lower panel). (**B**) Lung grafts extracted from WT recipients were soft with large lung volumes. Inflammation, injury and volume reduction were seen in grafts of KO recipients that showed consolidation on Micro-CT scan (lower panel). (**C**) Allograft consolidation on CT scan was graded from 1 to 5. Grades show significantly higher consolidation in the KO group compared to WT (*p* = 0.01). (**D**) Micro-CT lung volumes were analyzed in 7 WT and 6 KO mice in which the lung grafts were not completely consolidated. There is a trend towards lower volumes in the KO group. (**E**–**F**) Graft volume recovery was assessed by comparing the volumes at each time point to day 1 values. Volumes were partially recovered in the WT group but not in the KO group. ^*^P < 0.05, ^**^P < 0.005.

Micro CT scans were used to monitor the allografts. All grafts showed consolidation at day 7 post-transplant (Figure 1A). While 7/9 of the WT allografts cleared up by day 28 (Figure 1A, first row), 6/13 of the KO allografts remained partially (Figure 1A, second row) and 7/13 completely (Figure 1A, third row) consolidated on day 28. This radiological appearance correlated with the gross appearance of the lungs: explanted lungs from WT recipients were soft with larger lung volumes. PTX3-KO recipients showed shrunken and injured grafted lungs (Figure 1B).

For each CT scan, the allograft was given a grade from 1 to 5 to reflect the severity of consolidation. Based on this semi-quantitative schema, the KO allografts had significantly more severe consolidation that persisted until day 28, compared to the WT group where the consolidation resolved (Figure 1C).

For a more quantitative assessment of the CT scans, we measured the actual graft volume. Since 2/9 WT grafts and 7/13 KO grafts were completely consolidated, could not be distinguished from the surrounding soft tissue and chest wall, lung volume was quantified only on the remaining grafts with computer software. The KO group showed a trend towards lower volumes compared to the WT group, even though this difference was not statistically significant when analyzed using a 2-way ANOVA (Figure 1D). We then asked the question whether lung volume recovery differed between the KO and WT groups, by comparing lung volumes to the day 1 baseline. In the WT group, lung volumes were significantly lower on day 7 compared to day 1; however, the volumes recovered from days 14 to 28 (Figure 1E). In contrast, in the KO group, volumes on days 7, 14, 21, and 28 were all significantly lower than day 1 (Figure 1F). Hence, even after the exclusion of the most severely affected allografts from the KO group, there was still a difference in lung volume recovery between the KO and WT groups.

### Increased lung fibrosis and incidence of bronchiolitis obliterans in KO mice

**Figure 2:**
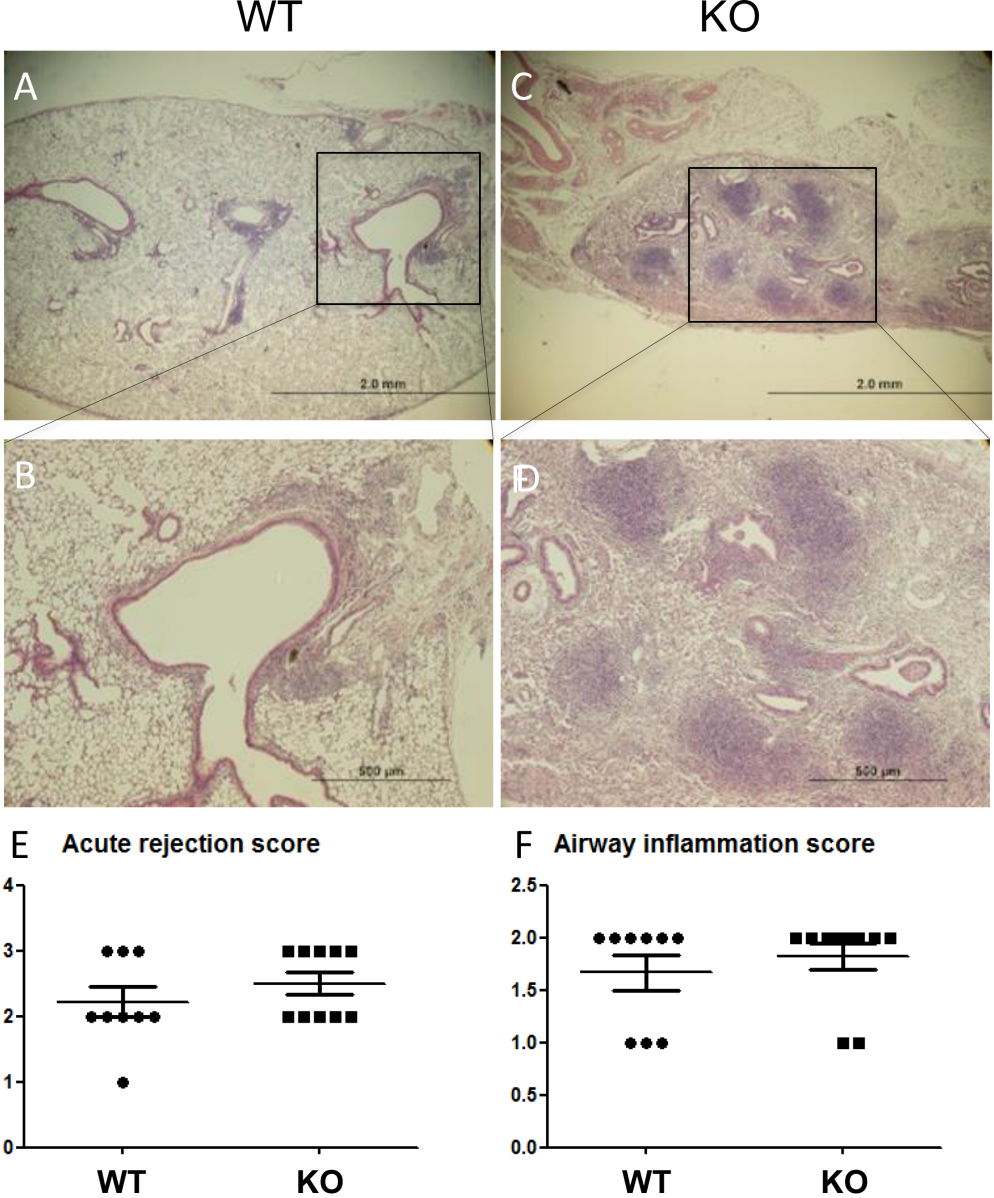
No difference in acute rejection grades between lung grafts of KO vs WT mice. (**A** and **C**) Low magnification H&E images. (**B** and **D**) Enlarged images from selected areas. Acute rejection was graded based on ISHLT criteria in *n* = 9 WT recipients and *n* = 10 KO recipients (lungs from 3 KO recipients had such extensive fibrosis that acute rejection was not gradable). (**E**) No significant difference in acute rejections scores. (**F**) No significant difference in airway (B-grade) inflammation scores.

**Figure 3:**
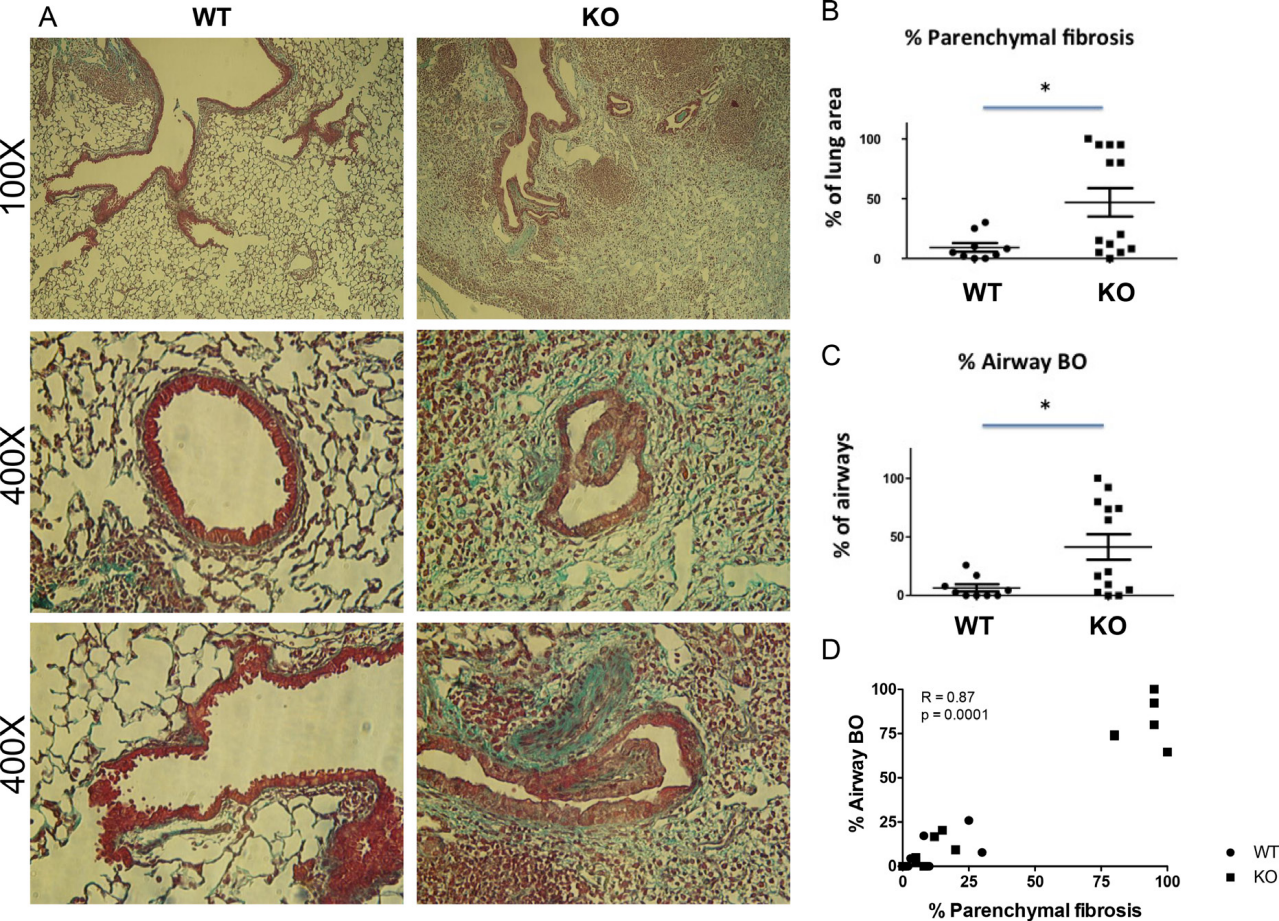
Increased parenchymal fibrosis and bronchiolitis obliterans (BO) in the lung grafts of KO mice (**A**) Examples of histological features of lung grafts imaged at 100X and 400X (two examples for 400X). (**B**) Semi-quantitative assessment shows increased % of parenchymal fibrosis in KO grafts (*n* = 9) compared to WT (*n* = 13) (*p* = 0.035) and (**C**) increased % of airways affected by BO lesions in KO grafts compared to WT (*p* = 0.031). ^*^P < 0.05. (**D**) The % of parenchymal fibrosis significantly correlates with the % of airways affected by BO (*P* = 0.0001).

Lung allograft histology was assessed 28 days after transplant. Acute rejection was graded according to the ISHLT guidelines [27]. Both acute rejection scores and airway inflammation scores were similar between the WT and KO groups (Figure 2). By contrast, parenchymal fibrosis, as assessed with Masson Trichrome staining was significantly higher in the KO group (Figure 3A, 3B, and Supplementary Figure 2). Moreover, the percentage of airways affected by BO was significantly higher in the KO group (Figure 3A, 3C). Furthermore, there was a strong correlation between the parenchymal fibrosis and airway BO (Figure 3D).

**Figure 4:**
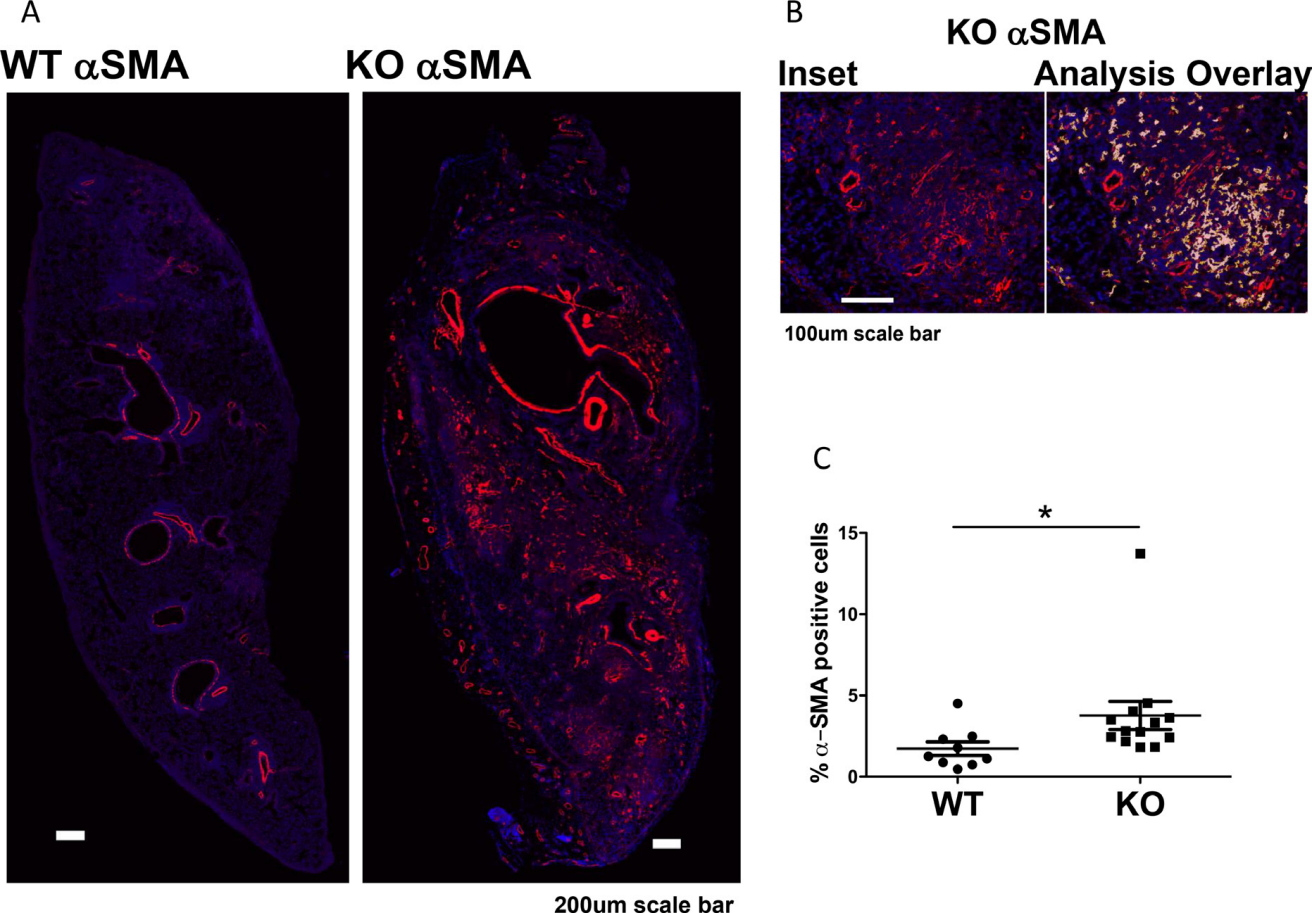
Increased myofibroblasts in lung grafts in KO mice (*n* = 13) compared to WT (*n* = 9) (**A**) Immunofluorescent staining of α-SMA of lung grafts. Original staining was converted to pseudocolors with computer software. (**B**) Inset showing an area in the KO lung with a matching image of the marker area overlay (with red, yellow, and white overlays for high, medium, and low intensity staining). Positive staining around the blood vessels and airways was excluded as vascular and airway smooth muscles. (**C**) Positive staining in the lung parenchyma was quantified and expressed as % of total lung area, showing increased α-SMA staining in KO grafts compared to WT (*P* = 0.0092). ^*^P < 0.05.

To determine whether the increased fibrosis was related to increased numbers of myofibroblasts, we performed immunofluorescent staining for α-SMA positive cells in the grafts. We excluded α-SMA positive smooth muscle cells around vessels and airways. Grafts in KO mice had more α-SMA positive staining (myofibroblasts) than grafts in WT mice (Figure 4).

### Increased lymphoid aggregation in lung grafts of KO mice

**Figure 5:**
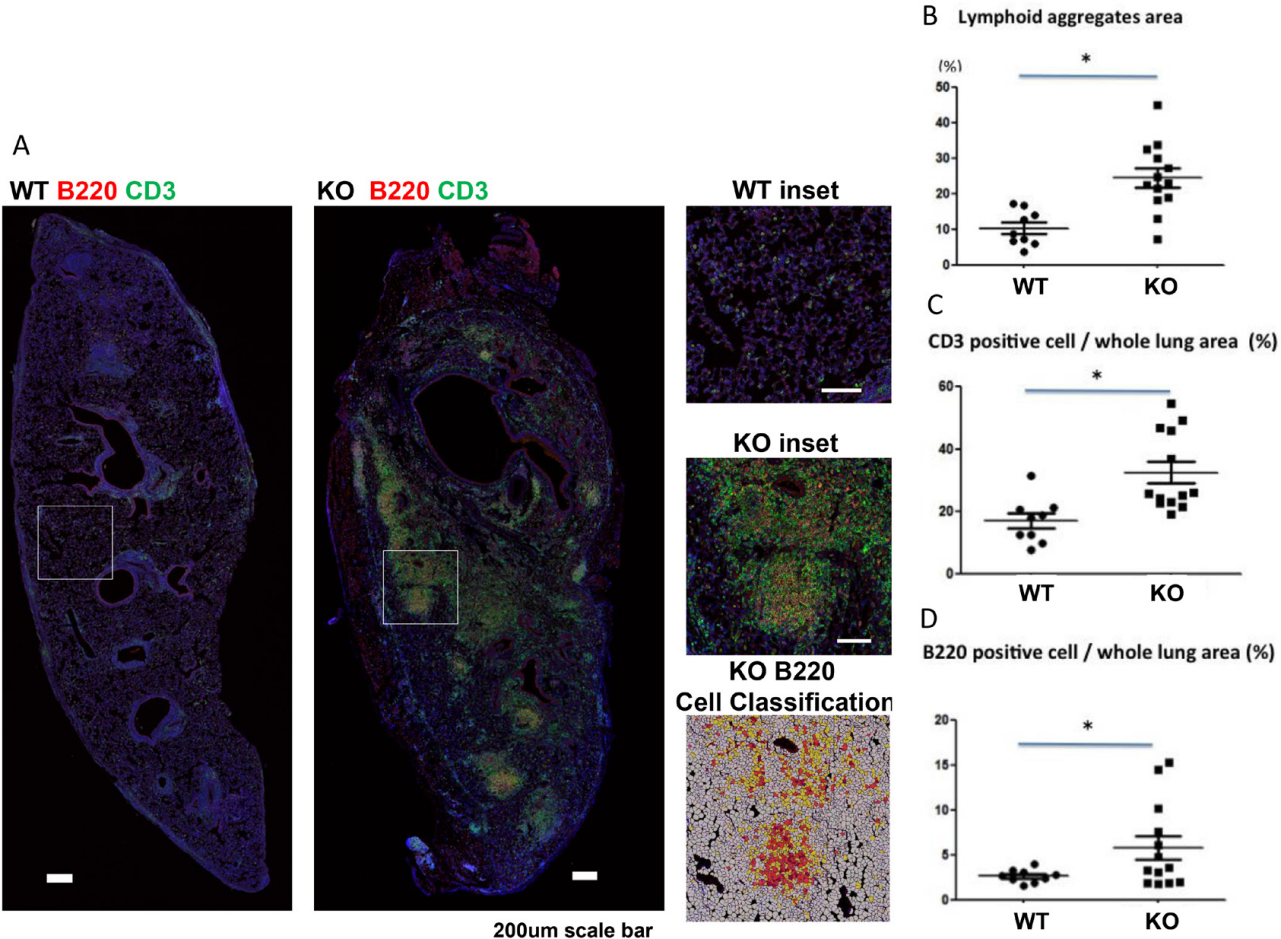
Increased lymphoid aggregates in lung grafts of KO (*n* = 9) compared to WT (*n* = 13) mice (**A**) Immunofluorescent staining of lymphoid aggregates with anti-CD3 (T cells) and anti-B220 (B cells) double staining and counterstained with DAPI for nuclei. Insets for both WT and KO lungs are shown. The cellular overlay matching the inset region with high (red), medium (orange), low (yellow), or negative (white) staining for B200 (the red channel) is shown. (**B**) Lymphoid aggregate areas (*P* < 0.05), (**C**) CD3 positive cells (*P* = 0.0013), and (**D**) B220 positive cells (*P* = 0.035) were increased in KO compared to WT grafts. ^*^P < 0.05.

Lymphoid infiltration and aggregation has been reported to contribute to the development of CLAD [3–6]. We examined lymphoid aggregates using CD3/B220 double immunofluorescent staining, and staining intensity was analyzed. KO mice had significantly larger lymphoid aggregates in the lung grafts than WT mice (Figure 5A, 5B). T cell (CD3 positive) and B cell (B220 positive) staining were also both significantly higher in the lung grafts of KO mice than WT mice (Figure 5C and 5D).

Neutrophils and macrophages were assessed in the lungs of mice at day 28 post transplant by immunofluorescence staining for myeloperoxidase and F4/80, respectively. The total numbers of cells identified by DAPI staining were significantly higher in the KO group, however, the numbers of neutrophils and macrophages were not significantly different between the KO and WT groups (Supplementary Figure 3).

### Suppression of cytokines in lung grafts of KO mice

**Figure 6:**
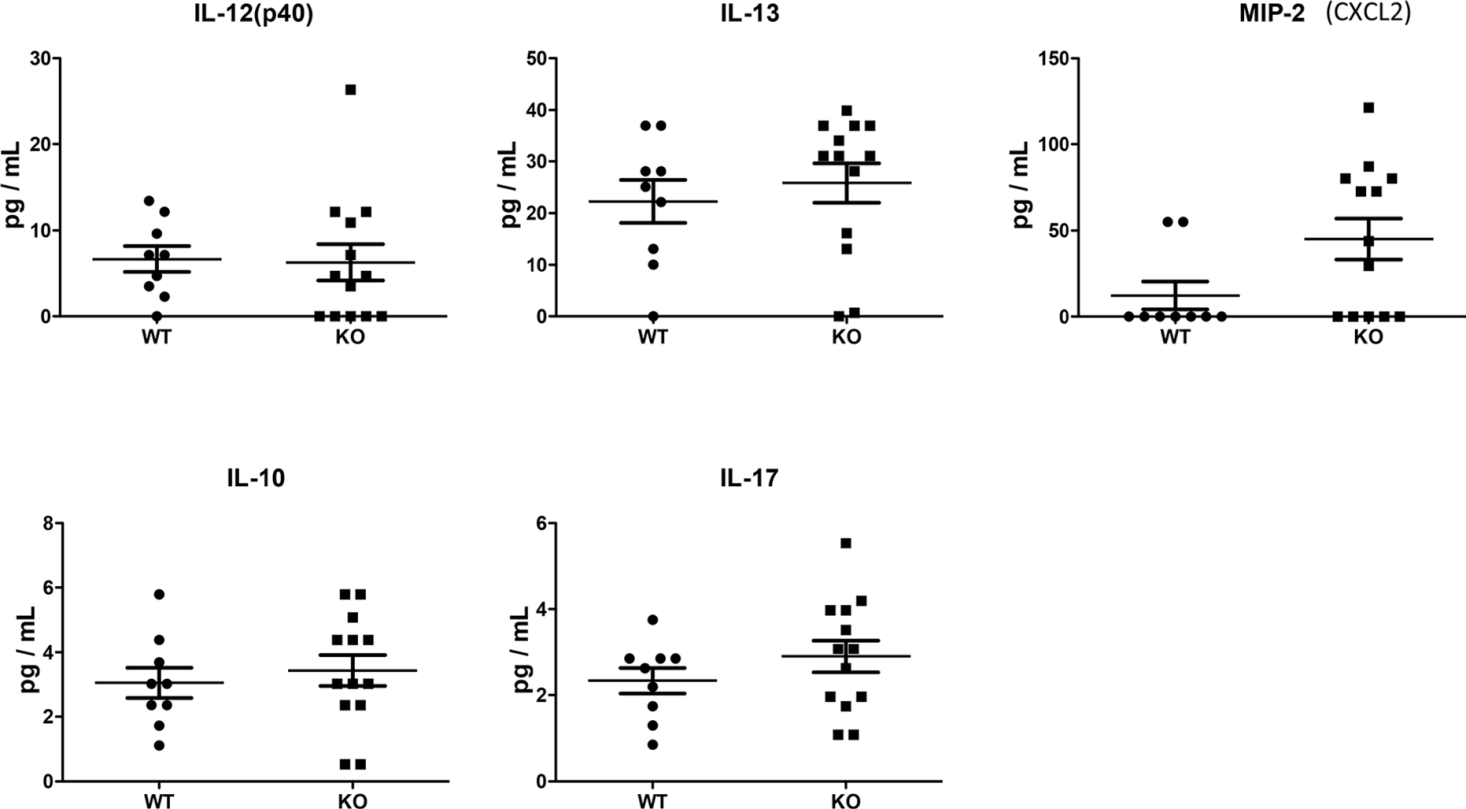
Cytokine and chemokine plasma levels at Day 28 were assessed using a microbead multiplex assay Of 22 cytokines and chemokines, 17 were below detection limits. No significant differences were found in IL-12, IL-13, IL-10, IL-17, or MIP-2 between WT (*n* = 9) and KO (*n* = 13) mice.

We assessed cytokines and chemokines in plasma collected on day 28, using a multiplex assay. For this exploratory analysis, a commercially-available 22-plex was chosen, as it captured common cytokines and chemokines previously shown to be involved in CLAD/BO pathogenesis. Among 22 cytokines and chemokines analyzed, 17 were below the detection limits. No significant differences between the two groups were seen in terms of IL-10, IL-12, IL-13, IL-17 and MIP-2 (CXCL2) in the plasma (Figure 6).

**Figure 7:**
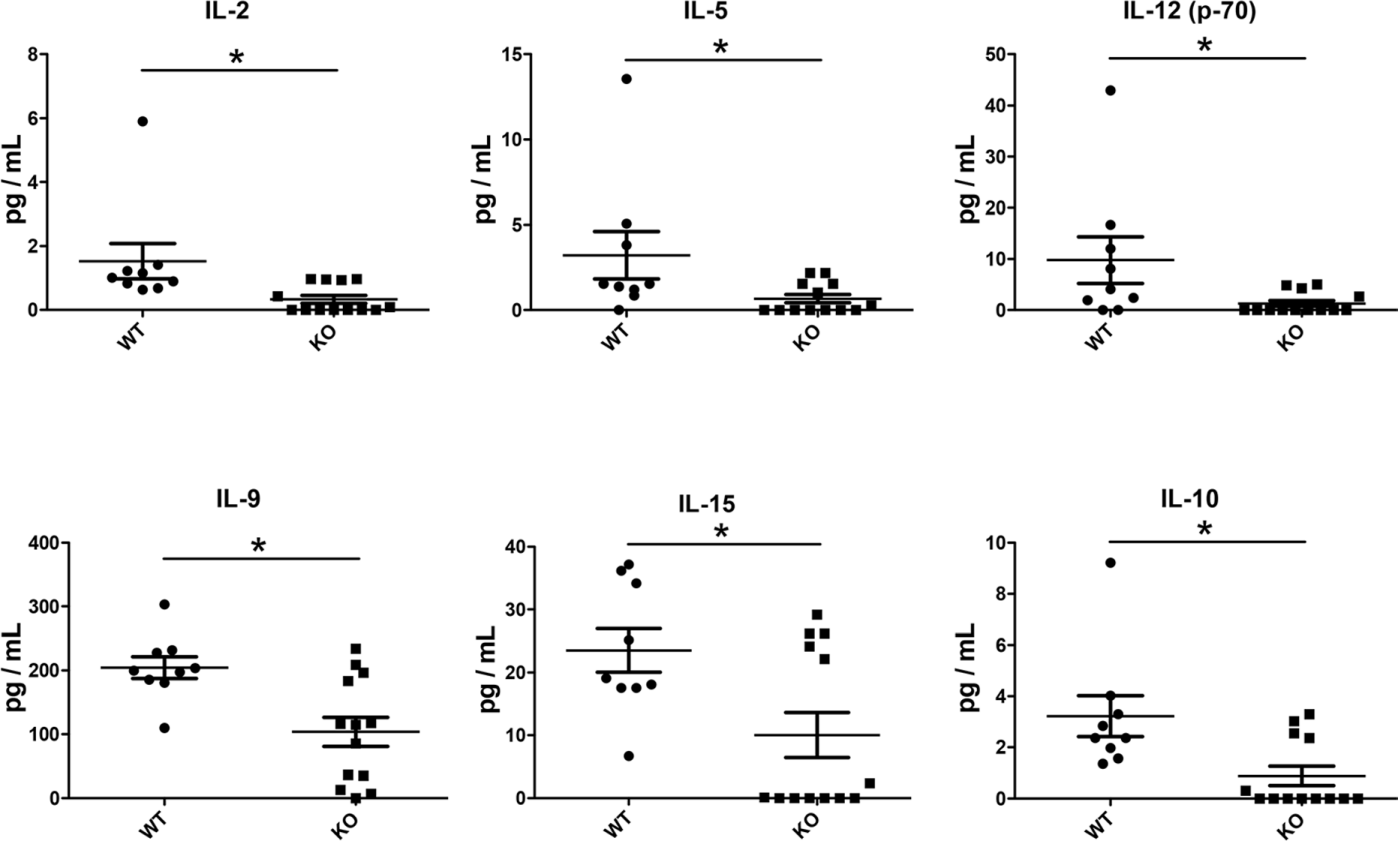
Reduced cytokine levels in lung grafts of KO (*n* = 13) compared to WT (*n* = 9) mice Cytokines and chemokines in the lung graft tissue lysates were analyzed with a multiplex microbead assay. The levels of a group of cytokines were significantly lower in the grafts of KO mice: IL-2 (*P* = 0.002), IL-5 (*P* = 0.043), IL-12 (*P* = 0.032), IL-9 (*P* = 0.016), IL-15 (*P* = 0.045), IL-10 (*P* = 0.015). ^*^P < 0.05.

We then analyzed cytokine and chemokine levels in the lung graft tissue. There were no significant differences in most cytokines analyzed, including IL-1α, IL-ß, TNFα, IFN-γ, IL-6, G-CSF, GM-CSF, IL-17 and IL-7 (Supplementary Figure 4). Similarly, there were no significant differences in the CC chemokines (CCL2, CCL3, CCL4, and CCL5) (Supplementary Figure 5A) or CXC chemokines (CXCL1, CXCL2 and CXCL10) (Supplementary Figure 5B) that were examined. Interestingly, however, IL-2, IL-15, IL-12, IL-5, IL-9, and IL-10, were significantly reduced in KO mice compared to WT mice (Figure 7).

## DISCUSSION

In the present study, using PTX3 KO mice, we demonstrate that the presence of PTX3 in lung transplant recipients plays a protective role against the development of chronic rejection-like pathology by reducing fibrotic lesions in small airways and lung parenchyma, which may be mediated through the inhibition of lymphocyte infiltration and aggregation in the lung.

### The mouse lung transplant model for CLAD studies

A major challenge to the study of mechanisms of development of CLAD is the lack of proper animal models [28]. The development of a mouse lung transplant model [26, 29] is a significant breakthrough in lung transplant research, which allows the use of transgenic mice for mechanistic studies. Fan et al., transplanting lungs from C57BL/10 H-2(b) mice into C57BL/6 mice, identified BO lesions at day 28 after lung transplantation [30]. We have recently used this model and demonstrated mixed airway and parenchymal lesions reminiscent of the pathological changes seen in CLAD [31]. The airway BO lesions appear to be a good correlate of airway fibrosis seen in patients with bronchiolitis obliterans syndrome [32]. In addition, the parenchymal fibrosis is reminiscent of the diffuse lung disease seen in patients with restrictive allograft syndrome [32]. In the present study, we again used C57BL/10 H-2(b) mice as donors, and the recipients were the F2 progeny of C57BL/6 and 129/SvEv/C57BL6/J mice. The lesions appear to be more severe than when C57BL/6 mice were used as recipients [31].

Transplantation of lungs from C57BL/10 to C57BL/6 mice results in acute rejection and BO in about 45–55% of cases [30]. Because both strains express the same H-2 alleles, the pathology is driven by an immune response directed at minor alloantigens. The PTX3-/- animals available for our studies were on a mixed 129/SvEv/C57BL6/J background. We generated F2 hybrids of these animals with C57BL/6 mice. Consequently, certain major alloantigens were also present in the recipients in variable quantities, suggesting there may have been some variability in the degree of alloreactivity from recipient to recipient. This may explain some of the pathological variability and the severe lesions in histological sections. Despite this variation, PTX3 deficiency clearly worsened early radiographic appearances of the allograft, day 28 pathology, and cellular infiltration.

One limitation of the present study was that we only have tissue and plasma samples collected at the end of 28 days. Studies from earlier time points would be helpful for understanding the role of PTX3 in the early stage of development of these pathological changes. However, mouse lung transplantation is a technically challenging protocol requiring extensive training and practice [33]. The mouse availability was further challenged by the infertility of female PTX3-/- mice [16, 18]; as a result, for this initial proof-of-principle study, we used only one time point for tissue collection. To partially compensate for this limitation, we used Micro-CT to follow changes in lung volume and lung appearance after transplantation. More than 50% of grafts from KO recipients showed consolidation; even the grafts without complete consolidation showed significantly less recovery of lung volumes. Micro-CT thus provides a means to monitor the status of the graft in a longitudinal fashion in individual animals. More detailed morphometric analyses of micro-CT scan may be of value and are presently being investigated. A major histocompatibility complex mismatch murine lung transplant model was recently developed, with B6D2F1/J (a cross between C57BL/6J and DBA/2J) (Haplotype H2b/d) mice as donors and DBA/2J (H2d) mice as recipients [34]. Since most of clinical lung transplants are major antigen mismatch, CLAD-related studies with this kind of models should be considered.

### Protective role of PTX3 in lung transplant recipients

Our results show that PTX3 in recipients is protective against development of airway and parenchymal fibrosis. As mentioned earlier, plasma PTX3 levels after lung transplantation were significantly associated with the development of PGD only in IPF patients, but not in COPD patients [13]. Similarly to IPF, CLAD is a fibrotic process, suggesting that PTX3 is linked to the fibrosis pathway. This is consistent with the clinical studies that report a lack of association between expression levels of PTX3 in plasma and COPD, which is an inflammatory rather than fibrotic process [35–37].

The most significant finding of this present study is the reduction in features of chronic rejection seen in WT recipients, as reflected by less lung tissue consolidation, less pulmonary fibrosis, especially occlusive airway lesions, and less myofibroblasts in the lung tissues. These results are in agreement with previous studies conducted with PTX3 deficient mice. For example, a lack of PTX3 aggravated post-ischemic acute kidney injury, followed by tubular atrophy, interstitial fibrosis, and kidney shrinkage, while injection of recombinant PTX3 up to 6 h after reperfusion prevented renal leukocyte recruitment and post-ischemic kidney injury [38]. PTX3 treatment accelerated recovery of kidney function after acute injury, by reducing collagen expression and α-SMA positivity, and inhibiting interstitial fibrosis [39]. PTX3 also plays a protective role in cardiomyocyte ischemia reperfusion injury with PTX3-deficient mice showing exacerbated heart damage, associated with a greater no-reflow area, increased neutrophil infiltration, decreased number of capillaries, and increased number of apoptotic cardiomyocytes [40]. PTX3 treatment ameliorated cardiomyocyte apoptosis and infiltration of neutrophils and macrophages and improved hemodynamic performance [41]. Recent studies suggest that PTX3 produced from bone marrow-derived cells plays a crucial role in cardiac protection against myocardial I/R injury by attenuating infiltration of neutrophils, generation of ROS and inflammatory cytokines [42]. These mechanisms may help explain the severe fibrotic lesions we observed in PTX3 deficient recipients after mouse lung transplantation. Exogenous PTX3 should be considered as a therapeutic option for CLAD in the future.

### PTX3 deficiency mediates lymphocytic infiltration

In the PTX3 KO recipients, dramatic lymphoid aggregates were seen in the grafts with increased numbers of T cells and B cells. Lymphocytic infiltration has been considered as a precursor of fibrous obliteration [3]. Lymphoid neogenesis has been found in animal models of BO [4], as well as in patients with BO or CLAD after lung transplantation [5]. The increased lymphoid aggregates in grafts of KO mice may be due to increased lymphoid infiltration and/or neogenesis of lymphoid tissues, which merits further investigation. We did not find differences in neutrophil or macrophage lung infiltration between KO and WT groups at day 28 post-transplant. The increased cellularity in the lung tissue, therefore, is mainly due to the lymphocytes. Earlier time points should be assessed in the future to further elucidate the mechanisms of PTX3 protection.

To explore the underlying mechanisms, we measured cytokines and chemokines using a broad multiplex assay. Circulating cytokines and chemokines were not significantly different between PTX3 KO and WT recipients. By contrast, in the lung grafts of PTX3 KO recipients, several cytokine levels were significantly lower than that in WT recipients. IL-2, along with IL-9 and IL-15, are members of one cytokine family, each has a four alpha helix bundle molecular structure; they share the gamma chain of IL-2 receptor for signaling. This family of cytokines functions as a master regulator to influence cell fate decisions, both priming for differentiation and helping to maintain a differentiated state [43]. Interestingly, the levels of IL-12, a cytokine that promotes Th1 differentiation, and IL-5 and IL-10, both are Th2 cytokines, were also reduced in allografts of KO recipinets. These changes appear to be specific, because other cytokines and chemokines examined did not show significant differences. IL-2 promotes Th1 differentiation by inducing IL-12Rβ2 and IL-12Rβ1, promotes Th2 differentiation by inducing IL-4Rα [43]. We speculate that even though T cells and B cells were higher in the allografts of KO recipients than that in WT animals, these cells may be under differentiated. Clearly, further investigation is required to understanding the role of PTX3 in transplantation related cytokine production and differentiation of lymphocytes in the grafts.

In summary, PTX3 produced by lung allograft recipients may help regulate allograft repair, preventing fibrosis in the airways and lung tissues, and preventing lymphoid aggregation in the lung grafts. Ample evidence from animal models has shown that exogenous PTX3 administration ameliorates tissue injury and improves organ function. The protective role of endogenous PTX3 demonstrated in the present study suggests that the therapeutic effects of exogenous PTX3 merit further investigation.

## MATERIALS AND METHODS

### Animals

PTX3-KO mice were generated by breeding ptx3+/− heterozygous animals (129/SvEv/C57BL6/J background from Dr. M. M. Matzuk, Baylor College of Medicine) with C57BL/6 mice to obtain F2 generation ptx3-/- homozygous animals [22, 44, 45]. Mice were genotyped by PCR with two pairs of primers (Supplementary Table 1) designed against murine PTX3 Exon 2 and human HPRT contained in the targeting vector (Supplementary Figure 1A) [46]. Examples of PCR genotyping were shown in Supplementary Figure 1B. Male PTX3-KO mice of 8 to 17 weeks of age and their male WT littermates were used as recipients of lung transplants. Male C57BL/10 of 8-13 weeks of age (Jackson Laboratory, Bar Harbor, ME, USA) were maintained on a standard diet and kept in a pathogen-free environment for at least one week, and then used as donors.

### Animal procedures: lung transplantation

Mouse orthortopic lung transplantation was conducted following the technique previously described [26]. All animals received care in compliance with the Guide to the Care and Use of Experimental Animals formulated by the Canadian Council on Animal Care. The Animal Care Committee of the Toronto General Hospital Research Institute approved the experimental protocol. After lung transplantation, recipient mice were housed in a pathogen-free environment and maintained on a standard diet without antibiotics and immunosuppressant until euthanized at day 28, and lung tissue and blood samples were collected.

### Micro-computer tomography

At Day 1, 7, 14, 21, 28 after lung transplantation, mice were lightly anesthetized by isoflurane inhalation and microcomputer tomography (micro-CT) was taken using GE Locus Ultra Micro CT (GE Healthcare, London, Canada) with 4 s anatomical scans to cover the whole lung [31]. The allograft appearance on CT scan was assigned a grade based on the amount of consolidation: 0 = clear, 1 = minimal consolidation, 2 = mild consolidation, 3 = moderate consolidation, 4 = severe consolidation, 5 = complete consolidation (where the lung cannot be distinguished from the surrounding chest wall). The images where the outline of the lungs could be reliably defined were used for further volumetric analyses. The obtained images were analyzed using MicroView 2.2 software (GE Healthcare) to measure 3D lung volume, using the following approach: The outlines of each lung were traced from approximately 10 sections, from the apex to the basal portion of the lung region. The software generated a 3D region of interest by interpolating between each manually drawn region of interest, reconstructing a 3D volume containing the lung. The graft lung volume was then calculated by measuring the volume of this region of interest [31].

### Lung tissue collection & histological staining

The inferior 1/8 of the lung allograft was clamped, cut off, snap-frozen in liquid nitrogen, and preserved at -80 °C for later protein analysis. To collect the rest of the lung tissue, the left atrium was cut, 0.9% saline was injected through the pulmonary artery (PA), and both lungs were perfused. Then, 4% paraformaldehyde was injected into the lung through the intubated trachea at 20 cmH_2_O. The heart and lungs were removed *en-bloc* and placed in 10% formalin, then transferred into 70% ethanol. Each lung graft was cut into 3 parts: upper, middle and lower. These sections were then paraffin-embedded, sectioned, and used for hematoxylin and eosin (H&E), or Masson Trichrome (MT) staining as described previously [31].

All histological assessments were performed in a blinded fashion. Acute rejection was graded based on ISHLT criteria [47]. Parenchymal fibrosis was graded as percent of lung tissue affected by parenchymal fibrosis (estimation made based on MT slides). Airway bronchiolitis obliterans (BO) was assessed based on high-power examination of MT slides: all airways and airways affected by (BO) were counted within all parts (upper, middle, and lower) of each lung sample. The percentage of airways affected by BO was then calculated.

### Immunofluorescence staining

Paraffin-embedded tissue sections were deparaffinized, and antigen retrieval was performed using boiling 0.01M citrate buffer (pH 6) for 20 min. Sections were blocked with 5% goat serum in Dako serum-free blocking reagent for 30 min. For α-smooth muscle actin (α-SMA) staining, sections were incubated overnight at 4°C with Cy3-conjugated α-SMA antibody (1:400)(Sigma-Aldrich, Oakville, Ontario, Canada). After washing with PBS-0.1% Tween, sections were counterstained with DAPI and mounted with Slow Fade mounting medium (Invitrogen, Waltham, MA, USA). For CD3/B220 staining, the sections were incubated overnight at 4°C with CD3 antibody (1:200)(DAKO, Mississauga, Ontario, Canada) and Biotin conjugated B220 antibody (1:250)(BD, Mississauga, Ontario, Canada). After washing, sections were incubated with goat anti-rabbit IgG Alexa Fluor^®^ 555 and Alexa Fluor^®^ 488 streptavidin (Molecular Probes, Eugene, OR) at the dilution of 1:400. Antibodies for myeloperoxidase (MPO) (1:400) and F4/80 (1:500)(Abcam, Cambridge, MA, USA) were used for neutrophil and macrophage staining, respectively. Goat anti-rabbit IgG Alexa Fluor^®^ 555 (Molecular Probes, Eugene, OR, USA) were used as the secondary antibody at the dilution of 1:400. Then the sections were counterstained with DAPI and mounted.

### Quantification of lymphoid aggregates area

For each lung graft, as described above, sections from the upper, middle and lower part were stained and the whole area of each section was assessed. Slides were digitized using a Tissue Scope confocal fluorescence slide scanner (Huron Technologies, Waterloo, Ontario, Canada) at 0.5 um per pixel resolution. Computer-aided image analysis was performed using Definiens Tissue Studio software platform (Definiens AG, Munich, Germany). The software was first used to segment the entire lung into two sub-regions composed of lymphoid aggregate areas and bulk lung tissue, based on the presence of CD3 and B220 positive cells in lymphoid aggregates. Following this, cellular segmentation was performed based on the presence of DAPI signal, which was used to identify all cells in the lymphoid aggregate and lung regions of interest. Thresholds for positive staining were set by observation of true positive cells present in the lymphoid aggregates, and background lung tissue, with three thresholds each set to classify the CD3 and B220 positive cells as high, middle, low and negative staining. High staining indicated robustly positive cells; medium staining indicated positive stained cells, and low indicated marginal degrees of staining, but not reaching the threshold of true positivity. This machine-learning analytic technique is more precise than manual cell counting and has been described previously [48]. The high and middle intensity positive cells were summed up and divided by the whole lung cell number in the same area (counter-stained with DAPI), to calculate a percentage of CD3 and B220 positive cells.

### α-SMA positive cell quantification

α-SMA positive cells include vascular and airway smooth muscle cells and myofibroblasts. α-SMA positive staining was analyzed with Definiens TissueStudio. Positive staining around vessels and bronchi were excluded from the analysis so as to focus primarily on myofibroblasts in the lung tissue itself, not the vascular and airway smooth muscles. As with CD3 and B220 analysis above, the myofibroblast signal was assessed (this time on a per-pixel basis) by thresholding to identify high, medium, low and negative signal. The staining intensity of positive areas was assessed by counting regions positive for each class of signal, shown in screenshots with red representing very high, yellow high and white medium intensity. These three intensity signals were included as positive staining: α-SMA positive pixel count (mm^2^) /total lung area (mm^2^) X 100%.

### Quantification of macrophages and neutrophils

Six samples each from KO and WT groups were available for quantification of macrophages and neutrophils. Six fields per mouse were randomly chosen and each of the fields consisted of 3 x 3 stitched fields captured at 200x magnification on a confocal microscope. Number of MPO and F4/80 positive cells and DAPI-stained nuclei were counted using ImageJ ver. 1.45r (National Institutes of Health, Bethesda, MD, USA). The average number of cells per high-power-field was quantified.

### Soluble protein measurement:

Blood was collected at the time of necropsy from the inferior vena cava and placed in heparinized tubes and centrifuged for 10 min. The plasma was stored at –80°C until further analysis. Cytokines and chemokines in the plasma and in graft lung tissue at day 28 after lung transplantation were measured using the Millipore Mouse Cytokine/Chemokine Magnetic Bead Panel (BD Bioscience, Mississauga, Ontario, Canada).

### Statistical analysis

Data are expressed as mean ± SEM. Binary comparisons were made using unpaired two-tailed non-parametric Mann-Whitney test. Comparison of longitudinal microCT data was first analyzed using a two-way analysis of variance test. Comparisons between multiple time points within the same group were further made using a one-way analysis of variance, with post hoc comparison using Bonferroni correction. Statistical analyses were performed using Prism (Graph Pad Software, La Jolla, CA).

## SUPPLEMENTARY MATERIALS FIGURES AND TABLE



## References

[1] Yusen RD, Christie JD, Edwards LB, Kucheryavaya AY, Benden C, Dipchand AI, Dobbels F, Kirk R, Lund LH, Rahmel AO, Stehlik J, International Society for Heart and Lung Transplantation (2013). The Registry of the International Society for Heart and Lung Transplantation: Thirtieth Adult Lung and Heart-Lung Transplant Report—2013; focus theme: age. J Heart Lung Transplant.

[2] Sato M, Liu M, Anraku M, Ogura T, D’Cruz G, Alman BA, Waddell TK, Kim E, Zhang L, Keshavjee S (2008). Allograft airway fibrosis in the pulmonary milieu: a disorder of tissue remodeling. Am J Transplant.

[3] Boehler A, Chamberlain D, Kesten S, Slutsky AS, Liu M, Keshavjee S (1997). Lymphocytic airway infiltration as a precursor to fibrous obliteration in a rat model of bronchiolitis obliterans. Transplantation.

[4] Sato M, Hirayama S, Hwang DM, Lara-Guerra H, Wagnetz D, Waddell TK, Liu M, Keshavjee S (2009). The role of intrapulmonary de novo lymphoid tissue in obliterative bronchiolitis after lung transplantation. J Immunol.

[5] Sato M, Hirayama S, Matsuda Y, Wagnetz D, Hwang DM, Guan Z, Liu M, Keshavjee S (2011). Stromal activation and formation of lymphoid-like stroma in chronic lung allograft dysfunction. Transplantation.

[6] Wagnetz D, Sato M, Hirayama S, Matsuda Y, Juvet SC, Yeung JC, Guan Z, Zhang L, Liu M, Waddell TK, Keshavjee S (2012). Rejection of tracheal allograft by intrapulmonary lymphoid neogenesis in the absence of secondary lymphoid organs. Transplantation.

[7] Andrade CF, Waddell TK, Keshavjee S, Liu M (2005). Innate immunity and organ transplantation: the potential role of toll-like receptors. Am J Transplant.

[8] Liu M (2006). Innate immunity and lung transplantation.

[9] Weber DJ, Wilkes DS (2013). The role of autoimmunity in obliterative bronchiolitis after lung transplantation. Am J Physiol Lung Cell Mol Physiol.

[10] Whitson BA, Prekker ME, Herrington CS, Whelan TP, Radosevich DM, Hertz MI, Dahlberg PS (2007). Primary graft dysfunction and long-term pulmonary function after lung transplantation. J Heart Lung Transplant.

[11] Huang HJ, Yusen RD, Meyers BF, Walter MJ, Mohanakumar T, Patterson GA, Trulock EP, Hachem RR (2008). Late primary graft dysfunction after lung transplantation and bronchiolitis obliterans syndrome. Am J Transplant.

[12] DerHovanessian A, Weigt SS, Palchevskiy V, Shino MY, Sayah DM, Gregson AL, Noble PW, Palmer SM, Fishbein MC, Kubak BM, Ardehali A, Ross DJ, Saggar R (2016). The Role of TGF-beta in the Association Between Primary Graft Dysfunction and Bronchiolitis Obliterans Syndrome. Am J Transplant.

[13] Diamond JM, Lederer DJ, Kawut SM, Lee J, Ahya VN, Bellamy S, Palmer SM, Lama VN, Bhorade S, Crespo M, Demissie E, Sonett J, Wille K, Lung Transplant Outcomes Group (2011). Elevated plasma long pentraxin-3 levels and primary graft dysfunction after lung transplantation for idiopathic pulmonary fibrosis. Am J Transplant.

[14] Diamond JM, Meyer NJ, Feng R, Rushefski M, Lederer DJ, Kawut SM, Lee JC, Cantu E, Shah RJ, Lama VN, Bhorade S, Crespo M, Demissie E, Lung Transplant Outcomes Group (2012). Variation in PTX3 is associated with primary graft dysfunction after lung transplantation. Am J Respir Crit Care Med.

[15] Imai N, Nishi S, Yoshita K, Ito Y, Osawa Y, Takahashi K, Nakagawa Y, Saito K, Takahashi K, Narita I (2012). Pentraxin-3 expression in acute renal allograft rejection. Clin Transplant.

[16] Garlanda C, Bottazzi B, Bastone A, Mantovani A (2005). Pentraxins at the crossroads between innate immunity, inflammation, matrix deposition, and female fertility. Annu Rev Immunol.

[17] Bottazzi B, Doni A, Garlanda C, Mantovani A (2010). An integrated view of humoral innate immunity: pentraxins as a paradigm. Annu Rev Immunol.

[18] He X, Han B, Liu M (2007). Long pentraxin 3 in pulmonary infection and acute lung injury. Am J Physiol Lung Cell Mol Physiol.

[19] Ketter P, Yu JJ, Cap AP, Forsthuber T, Arulanandam B (2016). Pentraxin 3: an immune modulator of infection and useful marker for disease severity assessment in sepsis. Expert Rev Clin Immunol.

[20] Muller B, Peri G, Doni A, Torri V, Landmann R, Bottazzi B, Mantovani A (2001). Circulating levels of the long pentraxin PTX3 correlate with severity of infection in critically ill patients. Crit Care Med.

[21] Mauri T, Coppadoro A, Bellani G, Bombino M, Patroniti N, Peri G, Mantovani A, Pesenti A (2008). Pentraxin 3 in acute respiratory distress syndrome: an early marker of severity. Crit Care Med.

[22] He X, Han B, Bai X, Zhang Y, Cypel M, Mura M, Keshavjee S, Liu M (2010). PTX3 as a potential biomarker of acute lung injury: supporting evidence from animal experimentation. Intensive Care Med.

[23] Bozza S, Bistoni F, Gaziano R, Pitzurra L, Zelante T, Bonifazi P, Perruccio K, Bellocchio S, Neri M, Iorio AM, Salvatori G, De Santis R, Calvitti M (2006). Pentraxin 3 protects from MCMV infection and reactivation through TLR sensing pathways leading to IRF3 activation. Blood.

[24] Gaziano R, Bozza S, Bellocchio S, Perruccio K, Montagnoli C, Pitzurra L, Salvatori G, De Santis R, Carminati P, Mantovani A, Romani L (2004). Anti-Aspergillus fumigatus efficacy of pentraxin 3 alone and in combination with antifungals. Antimicrob Agents Chemother.

[25] Cunha C, Aversa F, Lacerda JF, Busca A, Kurzai O, Grube M, Loffler J, Maertens JA, Bell AS, Inforzato A, Barbati E, Almeida B, Santos e Sousa P (2014). Genetic PTX3 deficiency and aspergillosis in stem-cell transplantation. N Engl J Med.

[26] Okazaki M, Krupnick AS, Kornfeld CG, Lai JM, Ritter JH, Richardson SB, Huang HJ, Das NA, Patterson GA, Gelman AE, Kreisel D (2007). A mouse model of orthotopic vascularized aerated lung transplantation. Am J Transplant.

[27] Stewart S, Fishbein MC, Snell GI, Berry GJ, Boehler A, Burke MM, Glanville A, Gould FK, Magro C, Marboe CC, McNeil KD, Reed EF, Reinsmoen NL (2007). Revision of the 1996 working formulation for the standardization of nomenclature in the diagnosis of lung rejection. J Heart Lung Transplant.

[28] Sato M, Keshavjee S, Liu M (2009). Translational research: animal models of obliterative bronchiolitis after lung transplantation. Am J Transplant.

[29] Krupnick AS, Lin X, Li W, Okazaki M, Lai J, Sugimoto S, Richardson SB, Kornfeld CG, Garbow JR, Patterson GA, Gelman AE, Kreisel D (2009). Orthotopic mouse lung transplantation as experimental methodology to study transplant and tumor biology. Nat Protoc.

[30] Fan L, Benson HL, Vittal R, Mickler EA, Presson R, Fisher AJ, Cummings OW, Heidler KM, Keller MR, Burlingham WJ, Wilkes DS (2011). Neutralizing IL-17 prevents obliterative bronchiolitis in murine orthotopic lung transplantation. Am J Transplant.

[31] Oishi H, Martinu T, Sato M, Matsuda Y, Hirayama S, Juvet SC, Guan Z, Saito T, Cypel M, Hwang DM, Keller TL, Whitman MR, Liu M, Keshavjee S (2016). Halofuginone treatment reduces interleukin-17A and ameliorates features of chronic lung allograft dysfunction in a mouse orthotopic lung transplant model. J Heart Lung Transplant.

[32] Ofek E, Sato M, Saito T, Wagnetz U, Roberts HC, Chaparro C, Waddell TK, Singer LG, Hutcheon MA, Keshavjee S, Hwang DM (2013). Restrictive allograft syndrome post lung transplantation is characterized by pleuroparenchymal fibroelastosis. Mod Pathol.

[33] Tsushima Y, Jang JH, Wurnig MC, Boss A, Suzuki K, Weder W, Jungraithmayr W (2013). Mastering mouse lung transplantation from scratch--a track record. J Surg Res.

[34] Mimura T, Walker N, Aoki Y, Manning CM, Murdock BJ, Myers JL, Lagstein A, Osterholzer JJ, Lama VN (2015). Local origin of mesenchymal cells in a murine orthotopic lung transplantation model of bronchiolitis obliterans. Am J Pathol.

[35] Van Pottelberge GR, Bracke KR, Pauwels NS, Vermassen FE, Joos GF, Brusselle GG (2012). COPD is associated with reduced pulmonary interstitial expression of pentraxin-3. Eur Respir J.

[36] Schwingel FL, Pizzichini E, Kleveston T, Morato EF, Pinheiro JT, Steidle LJ, Dal-Pizzol F, Rocha CC, Pizzichini MM (2015). Pentraxin 3 sputum levels differ in patients with chronic obstructive pulmonary disease vs asthma. Ann Allergy Asthma Immunol.

[37] Duran L, Unsal M, Yardan T, Kati C, Bedir A, Turkeli S, Ekiz M (2015). The Evaluation of Serum Pentraxin-3 and High- Sensitivity C-Reactive Protein Levels in Patients with Acute Attack of COPD. Clin Lab.

[38] Lech M, Rommele C, Grobmayr R, Eka Susanti H, Kulkarni OP, Wang S, Grone HJ, Uhl B, Reichel C, Krombach F, Garlanda C, Mantovani A, Anders HJ (2013). Endogenous and exogenous pentraxin-3 limits postischemic acute and chronic kidney injury. Kidney Int.

[39] Xiao Y, Yang N, Zhang Q, Wang Y, Yang S, Liu Z (2014). Pentraxin 3 inhibits acute renal injury-induced interstitial fibrosis through suppression of IL-6/Stat3 pathway. Inflammation.

[40] Salio M, Chimenti S, De Angelis N, Molla F, Maina V, Nebuloni M, Pasqualini F, Latini R, Garlanda C, Mantovani A (2008). Cardioprotective function of the long pentraxin PTX3 in acute myocardial infarction. Circulation.

[41] Zhu H, Cui D, Liu K, Wang L, Huang L, Li J (2014). Long pentraxin PTX3 attenuates ischemia reperfusion injury in a cardiac transplantation model. Transpl Int.

[42] Shimizu T, Suzuki S, Sato A, Nakamura Y, Ikeda K, Saitoh S, Misaka S, Shishido T, Kubota I, Takeishi Y (2015). Cardio-protective effects of pentraxin 3 produced from bone marrow-derived cells against ischemia/reperfusion injury. J Mol Cell Cardiol.

[43] Liao W, Lin JX, Leonard WJ (2011). IL-2 family cytokines: new insights into the complex roles of IL-2 as a broad regulator of T helper cell differentiation. Curr Opin Immunol.

[44] Han B, Haitsma JJ, Zhang Y, Bai X, Rubacha M, Keshavjee S, Zhang H, Liu M (2011). Long pentraxin PTX3 deficiency worsens LPS-induced acute lung injury. Intensive Care Med.

[45] Han B, Ma X, Zhang J, Zhang Y, Bai X, Hwang DM, Keshavjee S, Levy GA, McGilvray I, Liu M (2012). Protective effects of long pentraxin PTX3 on lung injury in a severe acute respiratory syndrome model in mice. Lab Invest.

[46] Varani S, Elvin JA, Yan C, DeMayo J, DeMayo FJ, Horton HF, Byrne MC, Matzuk MM (2002). Knockout of pentraxin 3, a downstream target of growth differentiation factor-9, causes female subfertility. Mol Endocrinol.

[47] Yousem SA, Berry GJ, Cagle PT, Chamberlain D, Husain AN, Hruban RH, Marchevsky A, Ohori NP, Ritter J, Stewart S, Tazelaar HD (1996). Revision of the 1990 working formulation for the classification of pulmonary allograft rejection: Lung Rejection Study Group. J Heart Lung Transplant.

[48] Shiah YJ, Tharmapalan P, Casey AE, Joshi PA, McKee TD, Jackson HW, Beristain AG, Chan-Seng-Yue MA, Bader GD, Lydon JP, Waterhouse PD, Boutros PC, Khokha R (2015). A Progesterone-CXCR4 Axis Controls Mammary Progenitor Cell Fate in the Adult Gland. Stem Cell Reports.

