# Supplementary material for: Pentraxin 3 deficiency enhances features of chronic rejection in a mouse orthotopic lung transplantation model
Source: Oncotarget. 2018 Jan 3;9(9):8489–501. doi: 10.18632/oncotarget.23902 (PMC5823599; doi:10.18632/oncotarget.23902)
Supplement: Supplementary file 1 [file oncotarget-09-8489-s001.pdf]

Pentraxin 3 deficiency enhances features of chronic rejection in a mouse orthotopic lung transplantation model

SUPPLEMENTARY MATERIALS

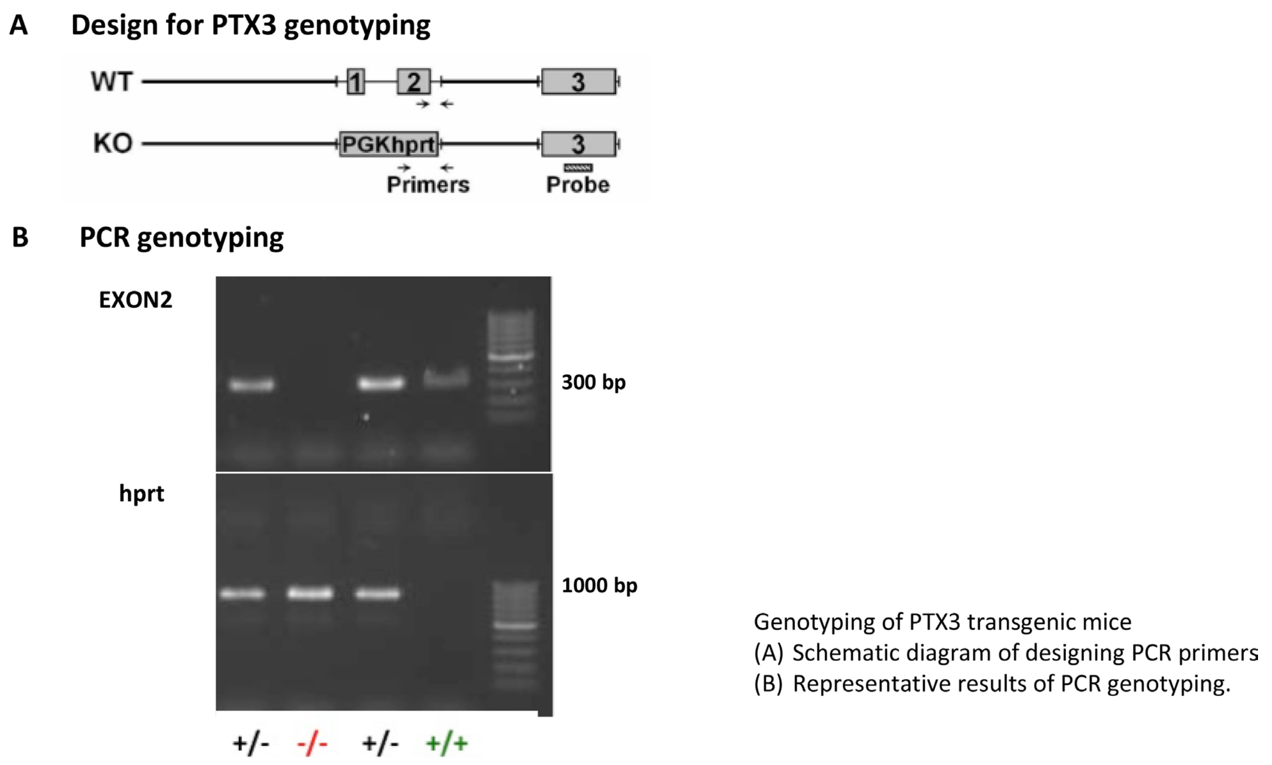

Supplementary Figure 1: Genotyping of PTX3 KO mice. (**A**) Schematic diagram of design of PCR primers for genotyping. (**B**) Example of PCR blots for genotyping.

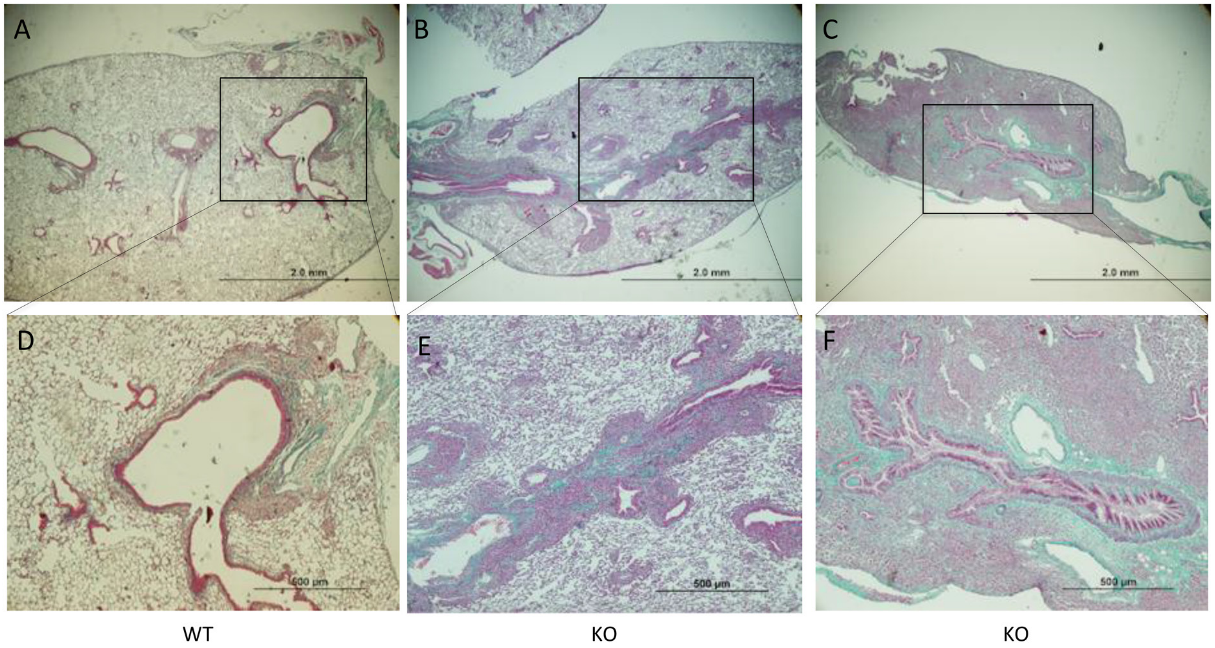

Supplementary Figure 2: Masson Trichrome staining on Day 28 after left lung transplantation. (**A–C**) x40 original magnification. (**D–F**) x100 original magnification. (A, D) Lung graft from WT recipient; (B, E) graft from KO recipient with less lung consolidation on CT; and (C, F) graft from KO recipient with severe lung consolidation based on Micro-CT.

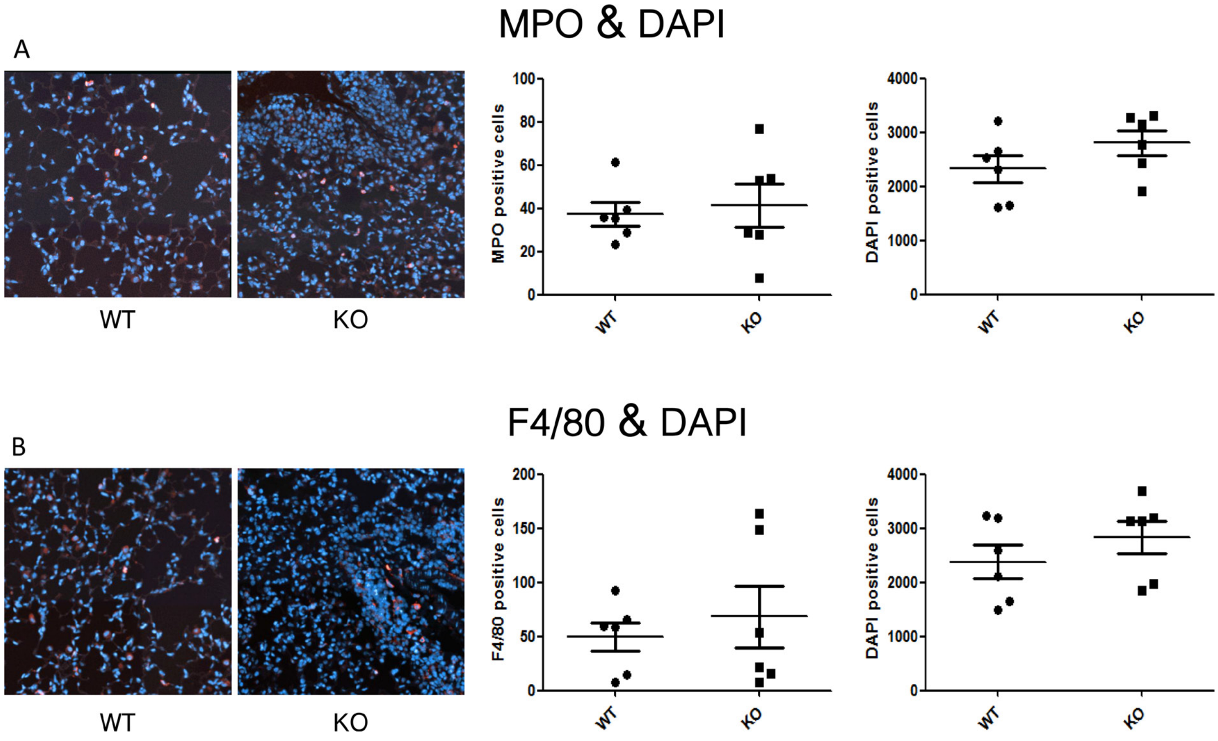

Supplementary Figure 3: No differences in neutrophil and macrophage infiltration in the lung tissues. Neutrophils (**A**) and macrophages (**B**) were stained with antibody against MPO or F4/80, respectively, and counterstained with DAPI for cell nuclei. There was no difference in the % of MPO positive cells or F4/80 positive cells, or total number of cells, between the two groups.

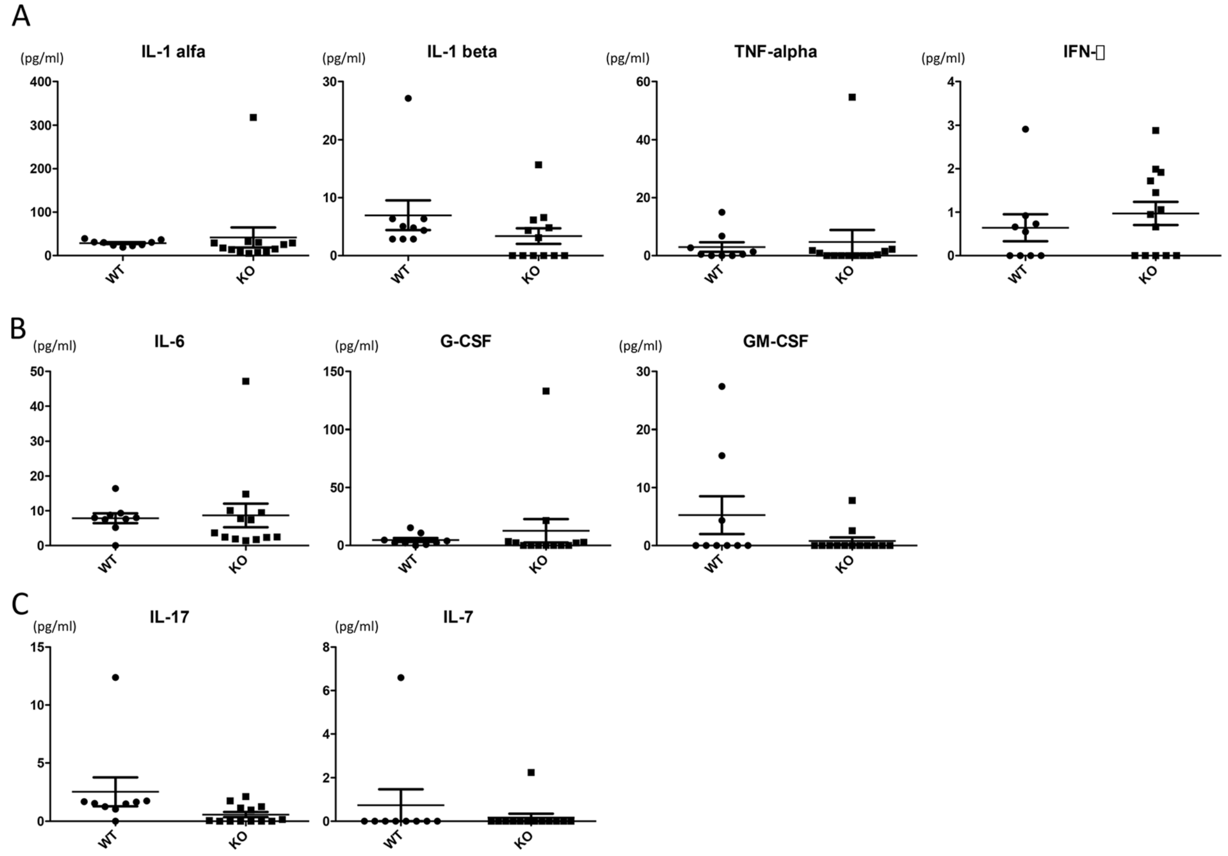

Supplementary Figure 4: No significant differences in multiple cytokine levels in lung graft tissue lysates. (**A**) Early response cytokines (IL-1α, IL-1β, TNFα, and IFNγ); (**B**) IL-6, G-CSF, GM-CSF; and (**C**) IL-17, IL-7.

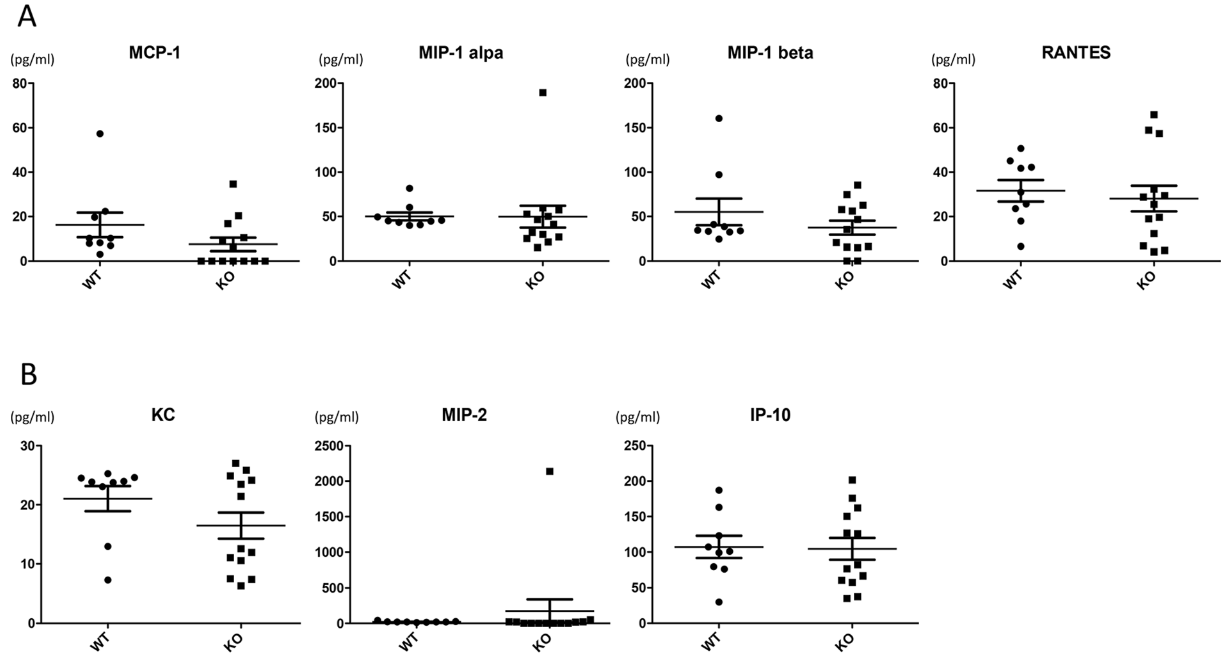

Supplementary Figure 5: No significant differences in multiple chemokine levels in lung graft tissue lysates. (**A**) CC chemokines: MCP-1 (CCL2), MIP-1 alpha (CCL3), MIP-1beta (CCL4) and RANTES (CCL5); (**B**) CXC chemokines: KC (CXCL1), MIP-2 (CXCL2) and IP-10 (CXCL10).

Supplementary Table 1: PCR primer sequences for PTX3 transgenic mouse genotyping

Targets	Forward primer (5′–3′)	Reversse primer (5′–3′)
Murine PTX3 exon 2	ACCTGAGCGCCGTGCAGA	TGTTAAACACCTGCCAATGG
Human HPRT	ACCCCACGAAGTGTTGGATA	TGTTAAACACCTGCCAATGG
